# Traumatic lumbar spondylolisthesis

**DOI:** 10.12669/pjms.291.2593

**Published:** 2013

**Authors:** Shujie Tang

**Affiliations:** Shujie Tang, MD, PhD, Department of Traditional Chinese Medicine, Medical School, Jinan University, Guangzhou, 510632, China.

**Keywords:** Traumatic lumbar spondylolisthesis, Posterior lumbar interbody fusion, Reduction, Decompression, Fixation

## Abstract

Traumatic lumbar spondylolisthesis is a rare lesion and frequently noted in patients with multiple traumatic injuries. We report one case of L5 traumatic spondylolisthesis, which obtained successful decompression, reduction, interbody fusion and fixation by posterior lumbar interbody fusion, and got satisfactory outcome. We recommend early decompression, reduction, interbody fusion and fixation with posterior instrumentation to obtain the recovery of neurological function and stability of the spine.

## Introduction

 Traumatic lumbar spondylolisthesis is an uncommon injury^1^ and its mechanism and treatment remain controversial. A combination of multiple forces may be responsible for the occurrence of the trauma^2^ and in English literatures, the injury was reported to be treated using different surgical approaches. We describe a patient with a traumatic spondylolisthesis of L5. Reduction, decompression and fixation were performed using posterior approach and at the last follow-up the case got a satisfactory result.

## Case Report

 A 41-year-old man was involved in a work-related accident and his low back was caught and crashed by machine. After he was released the patient was conscious and had motor weakness of grade 3/5 of the iliopsoas, quadriceps and tibialis anterior on both sides. There was slight sensory loss in both legs. There was no disturbance of urinary or bowel functions.

**Fig.1:**
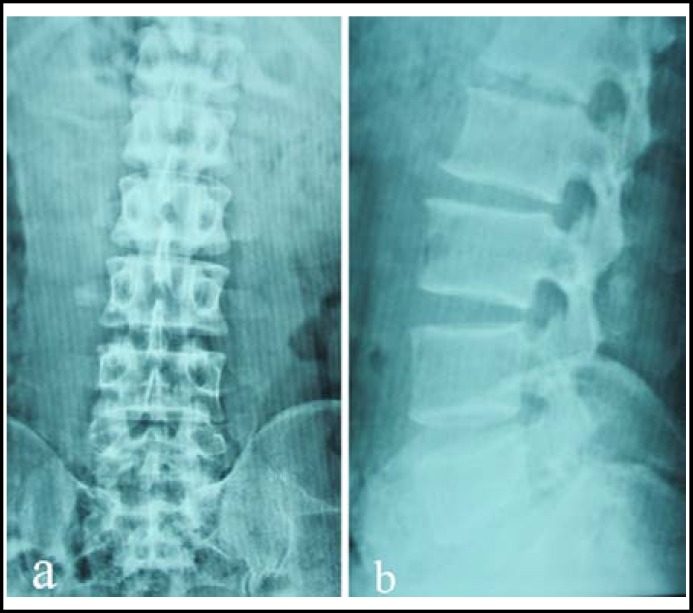
Preoperative X-radiographs (a. anteroposterior view and b. lateral view) displaying traumatic spondylolisthesis of L5 on S1.

**Fig.2:**
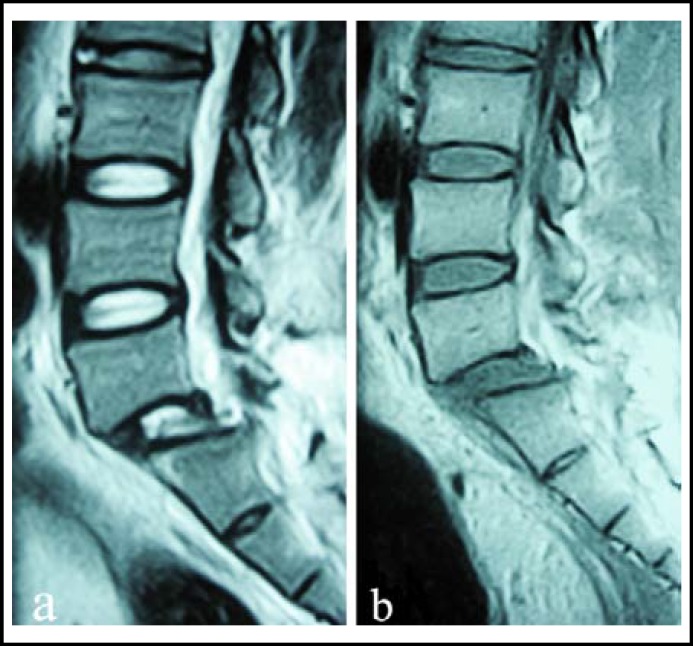
Sagittal magnetic resonance imaging (a. T2-weighted image and b. T1-weighted image) showing anterior displacement of L5 on S1with rupture of L5 intervertebral disc.

 The patient was admitted into trauma department of our hospital and X-ray examination (Fig.1) revealed traumatic lumbar spondylolisthesis of L5 on S1, fracture of left L1-3 transverse processes, bilateral fracture of transverse process and spinous process of L4, and fracture of spinous process of L5. MRI revealed L5 spondylolisthesis and disruption of L5 intervertebral disc (Fig.2). 

 Two days later, the patient underwent surgery in prone position using a standard posterior midline approach. At operation fracture of spinous process of L4-5, bilateral fracture-dislocation of L5 inferior facets, disruption of interspinal ligaments and flaval ligaments of L5-S1, disruption of annular fibrosus of L5 intervertebral disc were confirmed. Decompression, L5 disc excision and reduction were done followed by internal fixation using pedicle screws and rods from L5–S1. Two PEEK cages were inserted with autologous corticocancellous bone grafts. The operation lasted 89 minutes, the blood loss was about 200ml and no complications occurred intraoperatively.

**Fig.3:**
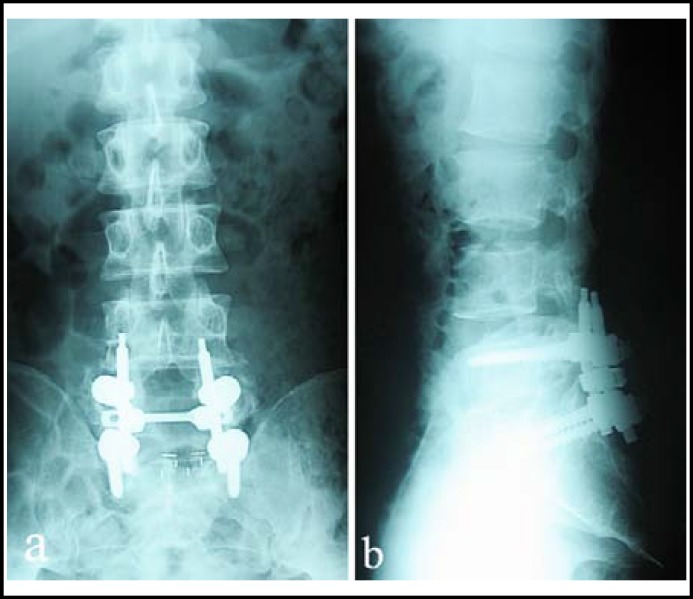
Two-year follow-up radiographs

 Postoperatively, radiographs revealed restoration of lumbar alignment and functional exercises were performed. He focused actively on the training of muscle groups of lower extremities. At three weeks after operation, his strength improved to 5/5 in lower extremities and his cutaneous sensation recovered completely. Two years after procedure, at the final follow-up, the patient had no low back pain or leg pain and radiographs (Fig.3) showed maintenance of the reduction and solid fusion with no breakage of instrumentations. He was satisfied with the outcome.

## Discussion

 Five types of spondylolisthesis are reported in literatures including dysplastic, isthmic, degenerative, traumatic and pathologic spondylolisthesis. Traumatic spondylolisthesis is usually accompanied by a fracture of the posterior elements, which result in instability and listhesis.^1^

Traumatic lumbar spondylolisthesis is uncommon lesion usually secondary to violent trauma. Among these injuries there were characteristic concomitant transverse processes fracture- of adjacent segments and according to the points of Herron^4^ and Roche PH^6^ the presence of transverse process fracture must result in the suspicion of associated traumatic lesions of the lumbosacral joint. Our case also suffered from fractures of transverse processes, which support the above viewpoint. However, the transverse process fracture is not the necessary sign associated with this kind of injury.^7^^,^^8^

 The mechanism of traumatic lumbar spondylolisthesis is complex and controversial. Some authors supported that hyperextension stress^7^, hyperflexion and compression stress^9^, or tangential force^9^ may be responsible for the occurrence of the trauma. It is challenging to propose an exact mechanism when the injury is complicated and severe. In our opinion, as the case had fracture of facet joints, the vectors of compression or axial translation may be the cause of the injury. Subsequently, we suggest the main factor is combination of tangential force and compression force.

 Most authors tend to treat the type of trauma by surgery, which can be performed using anterior, posterior or anterioposterior approach. It is essential to restore normal lumbar alignment, decompress the nerve structures and stabilize the lumbar spine, using open reduction and rigid fixation. In addition, clear evidence of disc disruption can be found on preoperative MRI in our case, so we treated the case using posterior lumbar interbody fusion and the case obtained acceptable reduction and stabilization.
